# Plumieride as a novel anti-fungal and anti-inflammatory iridoid against superficial candidiasis in mice

**DOI:** 10.1186/s12906-024-04508-z

**Published:** 2024-06-10

**Authors:** Riham A. El-Shiekh, Meselhy Rageb Meselhy, Rana Elshimy, Marwa A. Ibrahim, Merhan E. Ali, Eman I. Hassanen

**Affiliations:** 1 Department of Pharmacognosy, Faculty of Pharmacy, Cairo University, Cairo, 11562 Egypt; 2 Department of Microbiology and Immunology, Faculty of Pharmacy, Ahram Canadian University, Giza, 12573 Egypt; 3 Department of Microbiology and Immunology, Egyptian Drug Authority, Cairo, 15301 Egypt; 4 Department of Biochemistry and Molecular Biology, Faculty of Veterinary Medicine, Cairo University, Giza, 12211 Egypt; 5 Department of Pathology, Faculty of Veterinary Medicine, Cairo University, Giza, 12211 Egypt

**Keywords:** *Candida albicans*, Gene expression, Inflammation, Pathology, Plumieride

## Abstract

In the past few decades, there has been a notable rise in the occurrence of several types of candidiasis. *Candida albicans* is the most common cause of superficial fungal infections in humans. In this study, plumieride, one of the major iridoids from *Plumeria obtusa* L. leaves, was isolated and investigated for its potential against *Candida albicans* (CA)-induced dermatitis in mice. qRT-PCR was done to assess the impact of plumieride on the expression of the major virulence genes of CA. Five groups (*n* = 7) of adult male BALB/c mice were categorized into: group I: non-infected mice; group II: mice infected intradermally with 10^7^–10^8^ CFU/mL of CA; group III: CA-infected mice treated with standard fluconazole (50 mg/kg bwt.); group IV and V: CA-infected mice treated with plumieride (25- and 50 mg/kg. bwt., respectively). All the treatments were subcutaneously injected once a day for 3 days. Skin samples were collected on the 4th day post-inoculation to perform pathological, microbial, and molecular studies. The results of the in vitro study proved that plumieride has better antifungal activity than fluconazole, manifested by a wider zone of inhibition and a lower MIC. Plumieride also downregulated the expression of CA virulence genes (*ALS*1, *Plb*1, and *Hyr*1). CA-infected mice showed extensive dermatitis, confirmed by strong iNOS, TNF-α, IL-1β, and NF-κB genes or immune expressions. Whereas the treatment of CA-infected mice with plumieride significantly reduced the microscopic skin lesions and modulated the expression of all measured proinflammatory cytokines and inflammatory markers in a dose-dependent manner. Plumieride interfered with the expression of *C. albicans* virulence factors and modulated the inflammatory response in the skin of mice infected with CA.

**Supplementary Information:**

The online version contains supplementary material available at 10.1186/s12906-024-04508-z.

## Introduction

*Candida albicans* (CA) infection poses a serious clinical challenge in hospitalized patients, leading to a wide range of healthcare-associated fungal infections such as cutaneous candidiasis and fatal invasive candidiasis [1]. The treatment of CA infections requires addressing both candida virulence traits and antifungal resistance. CA can powerfully switch between budded yeast and filamentous forms (pseudohyphae, true hyphae) in a reversible manner, which contributes to its high pathogenicity in humans by facilitating proliferation and colonization in various tissues through its virulence traits [2, 3]. In the immune system, only phagocytic cells can kill *Candida in vitro*, making the resistance of CA to phagocyte killing a crucial virulence mechanism. This resistance is facilitated by the hypha-co-expressed gene *HYR1*, which not only mediates resistance to phagocytic killing in vitro but also modulates the fungal burden in tissue [4]. Another virulent trait is *Plb1*, which is secreted by CA during the invasion of the gastric mucosa; thus, downregulation of *Plb1* will result in attenuation of candida virulence [5]. On the other hand, CA has the ability to form complex biofilms consisting of various cell types, including yeast-form, pseudohyphae cells, and hyphae within an extracellular matrix. By regulating the expression of genes specific to hyphal growth, such as ALS1, it is possible to disrupt biofilm formation and consequently enhance the effectiveness of antifungal treatments [6].

*Plumeria obtusa* L. (family Apocynaceae) is a medicinally important plant that grows predominantly in tropical and subtropical regions. Traditionally, *P. obtusa* is used for gastrointestinal ailments, diabetes, malaria, fever, and pain. Decoction of the leaves is used to treat wounds and skin disorders [7]. The plant is reported to contain terpenes, sterols, iridoids (plumieride and derivatives), flavonoids, and phenolic acids [8]. The antimicrobial activity of *P. obtusa* L. has been well documented; the essential oil of *P. obtusa* flowers demonstrated antimicrobial activities against *Staphylococcus aureus*, *Bacillus cereus*, and *C. albicans* [9]. The biological activity could be attributed to the major class of compounds dominated by plumeria-type iridoids, i.e., plumieride, protoplumericin A, and plumieride acid, which displayed antimicrobial actions against some pathogenic bacteria and fungi in vitro [10]. Using LC/ESI-QToF, 37 compounds were identified in the methanolic extract of *P. obtusa* aerial parts (10 plumeria-type iridoids, 6 quinoline derivatives, 18 phenolics, 2 amino acids, and one fatty acid). We recently reported that the methanolic extract of the leaves and plumieride-type iridoids isolated thereof demonstrated promising antibacterial activity against multidrug-resistant Gram-negative strains; *Klebsiella pneumoniae* and Shiga-toxin producing *Escherichia coli* (STEC) [11]. According to the results of molecular docking studies, 13-*O*-coumaroyl plumieride was found to exhibit superior binding affinity for both iNOS and prostaglandin E synthase-1 target receptors and suggest its potential for targeting and modulating the activity of both enzymes [12].

The purpose of this study was to isolate plumieride from the leaves of *P. obtusa* cultivated in Egypt and evaluate its antifungal and anti-inflammatory activities against *C. albicans*-infected mice. The study aims to uncover the underlying processes of plumieride and examine the involvement of key factors such as iNOS, TNF-α, IL-1β, and NF-κB signaling in reducing the exaggerated inflammatory response in the skin.

## Materials and methods

### Plant material

*Plumeria obtusa L.* aerial parts were collected in October 2020 from Mazhar Botanic Garden in Giza, Egypt, and identified by Ms. Therese Labib, a botanical specialist and consultant at Orman Botanic Garden, Giza, Egypt. A voucher specimen (No. 3-07-2022) was deposited at the Pharmacognosy department at the Faculty of Pharmacy, Cairo University, Egypt.

### Plant extraction, fractionation, and isolation

**Fig. 1:**
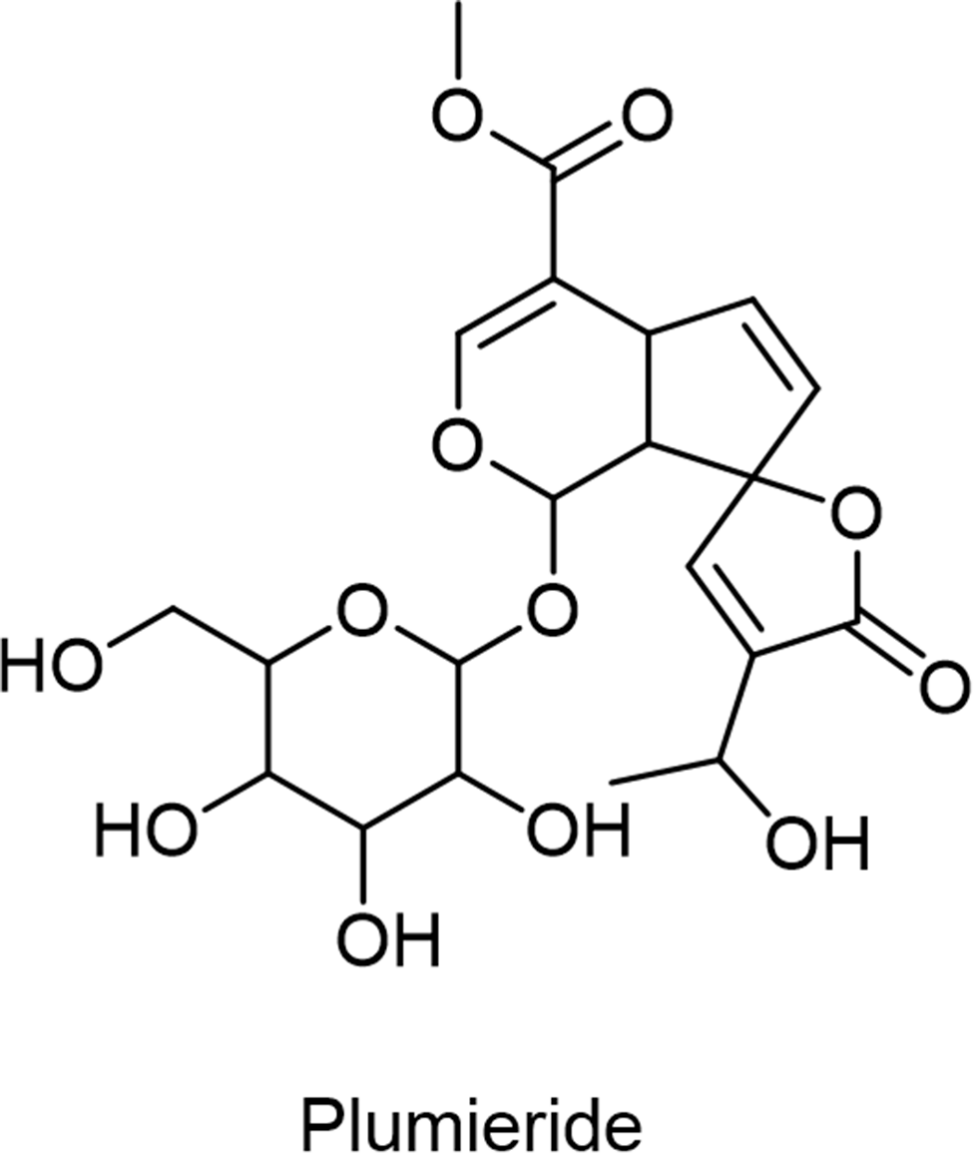
Chemical structure of plumieride

A sample (3.0 kg) of the plant material in powder form has been extracted by MeOH (37 L) at room temperature for 72 h using an Ultra Turrax T50 homogenizer (Germany). The extract was filtered and evaporated under low pressure to get 300 g of dry residue. A portion of the residue (200 g) was suspended in 500 mL of water and partitioned with dichloromethane (3 × 2 L). The water layer was applied to a Diaion HP-20 column (200 g), eluted with distilled water (2 L), 50% methanol-water (3 L), and 100% methanol (3 L), and fractions were evaporated under reduced pressure to yield a water fraction (29 g), a 50% methanol-water fraction (23 g), and a methanol fraction (M-F, 20 g), respectively. A part (2.5 g) of the M-F was fractionated by chromatography on a Si gel column (25 × 3 cm) using CH_2_Cl_2_-MeOH (8:2 v/v), and the test tubes from 41 to 57 (each of 15 mL) were collected and evaporated (390 mg), and then purified on a Si gel column (20 × 1.5 cm) using CH_2_Cl_2_-MeOH (6:4 v/v), followed by a RP-18 column using H_2_O-MeOH (7:3 *v*/v) to give 90 mg of pure compound. Its chemical structure was identified as plumieride (Fig. 1) by comparing ^1^H- and ^13^CNMR spectral data with those previously reported (Figs. S1 and S2) [11, 13].

### HPLC analysis

Chromatographic analysis was achieved on Agilent Technologies 1100 series HPLC system, equipped with autosampler injector, a quaternary pump, degasser G1322A and a diode-array detector (DAD). The separation was done on an Eclipse XDB-C18 column (150 × 4.6 mm, particle size 5 μm) with a C18 guard column (Phenomenex, Torrance, CA) and the column was operated at room temperature. The data acquisition and processing were done using Agilent Chemstation software. The methanolic fraction (2 mg/ mL) and the isolated compound (1 mg/ mL) were prepared in HPLC methanol and used as working solutions. Satisfactory separation was done at room temperature, within 15 min, by gradient elution using mixture of 0.3% phosphoric acid (A) and acetonitrile (B). A stepwise gradient elution program was adopted: 15-35% A (0–12 min), and 100% B (12–15 min). The flow rate was adjusted at 1 mL/min and the UV detector was set at 280 nm. Aliquots of 20 µL of each sample were directly injected into HPLC system (Fig. S3).

### Fungal strain isolation

Clinical *C. albicans* strain was isolated from female vaginal specimens from a tertiary care hospital lab. The isolate was enriched using a meat-peptone nutrient agar slant supplemented with 1% glucose. The agar slant was then incubated at 37 °C for 18–20 h. Yeast inoculum suspensions were prepared following the protocol outlined in CLSI M27-A2. In brief, five colonies with a diameter greater than 1 mm were selected for each isolate. These colonies were suspended in a 0.9% saline solution and adjusted to a final concentration of 0.5 McFarland standard using approximately 1–5 × 10^6^ CFU/mL. This suspension was either directly used for inoculating agar plates in the disc diffusion (DD) procedure or diluted for the microdilution (MD) procedure.

### Antifungal agents

Fluconazole (FZ), an antifungal agent obtained from SEDICO Pharmaceutical Co., Cairo, Egypt, was employed in the susceptibility tests. Moreover, FZ discs containing 25 µg obtained from Becton Dickinson in Sparks, MD, were utilized in the disc diffusion procedure. In the microdilution procedure, FZ was dissolved in distilled water to prepare a stock solution with a concentration of 6400 µg/mL. Two-fold serial dilutions ranging from 0.125 to 32 µg/mL were done [14].

### Antifungal susceptibility testing

#### Disc diffusion assay

To assess the antimicrobial activity of plumieride, an initial screening was conducted using the agar disc-diffusion method [15]. Positive controls were standard commercial antibiotic discs, FZ 25 g, while negative controls were discs impregnated with 0.2% DMSO. After 24 h of incubation at 37 °C, zones of inhibition surrounding the discs were detected and measured. The experiments were conducted in triplicate, and the full experiment was conducted twice.

#### Minimum inhibitory concentration (MIC)

The MIC was defined as the lowest drug concentration that inhibited the visible growth of a microorganism after overnight incubation when compared to a drug-free control well. Plumieride was dissolved in DMSO at a concentration of 2000 µg/mL, and different concentrations were made by two-fold serial dilution to yield dilutions ranging from 2000 µg/mL to 1.95 µg/mL. The substance is deemed highly active if the MIC is lower than 100 µg/mL, moderately active if the MIC ranges from 100 to 625 µg/mL, or weakly active if the MIC is higher than 625 µg/mL [16–19].

### Molecular identification for the isolated *C. Albicans*

#### Multiplex PCR for screening of *C. Albicans* virulence genes

DNA extraction was performed using the QIAamp DNA Mini kit (Qiagen, Germany, GmbH). Oligonucleotide primers were supplied from Metabion (Germany).

#### Differential real-time PCR for *C. Albicans* virulence gene expression

The total RNA extraction was performed following the “Enzymatic Lysis” procedure of QIAamp RNeasy Mini kit (Qiagen, Germany, GmbH) [20]. The primer sequences for the target genes (*SAP4*: Secreted aspartic peptidases encoding gene, *ALS1*: The agglutinin-like sequence gene, *HWP1*: Hyphal wall protein 1, *ALS3*: hypha-specific gene, *RAS1*: hyphal regulator gene, *Plb1*: the gene encoding the predominant phospholipase B secreted by *C. albicans*, *Hyr1*: hypha-specific genes, virulence factor that resists neutrophil killing) were collected in Table (1).

**Table 1:** Primers sequences and amplicon sizes for target genes of *C. albicans*

Target Gene	Primer sequence	Amplified segment (bp)	Reference
*SAP4*	GCT CTT GCT ATT GCT TTA TTA	394	[21]
	TAG GAA CCG TTA TTC TTA CA		
*ALS1*	GAC TAG TGA ACC AAC AAA TAC CAG A	318	[6]
	CCA GAA GAA ACA GCA GGT GA		
*HWP1*	ATG ACT CCA GCT GGT TC	572	
	TAG ATC AAG AAT GCA GC		
*ALS3*	CTGGACCACCAGGAAACACT	122	[22]
	ACCTGGAGGAGCAGTGAAAG		
*RAS1*	CCCAACTATTGAGGATTCTTATCGTAAA	106	
	TCTCATGGCCAGATATTCTTCTTG		
*Plb1*	ATGATTTTGCATCATTTG	751	[5]
	AGTATCTGGAGCTCTACC		
*Hyr1*	CGTCAACCTGACTGTTACATC	243	[4]
	TCTACGGTGGTATGTGGAAC		

### Animals and experimental design

This experimental design was conducted following the instructions of the institutional animal care and use committee of Cairo University (approval number: Vet Cu 08072023690).

Thirty-five adult male BALB/c mice with an average weight of 35 g were obtained from the Holding Company for Biological Products and Vaccines (VACSERA), Egypt. Every mouse was kept in a well-ventilated plastic cage with seven mice per cage, and they were exposed to 12 h of light each day. During the trial, they were given ad libitum access to tap water and were fed on dry commercial standard pellets. To make sure they were healthy, they were allowed to acclimatize to their surroundings for two weeks before the experiment started.

Every mouse had its back fur shaved, and the visible skin was sterilized with a solution of 10% povidone/iodine and 70% ethyl alcohol. The growing phase of CA cells was harvested, cleaned, and resuspended in saline. Then, in all except group 1, which was maintained as a negative control group, 50 mL of the suspension (10^7^–10^8^ CFU/mL) was deep intradermally injected into the mice’s backs [23, 24]. One hour after infection, mice were randomly divided into 5 groups (*n* = 7 per group) and received the following treatments subcutaneously into the infected area once a day for 3 days:

#### Group 1:

Negative control group given sterile normal saline without Candida infection.

#### Group 2:

CA-infected group given sterile normal saline.

#### Group 3:

CA-infected group treated with FZ (50 mg/kg).

#### Group 4:

CA-infected group treated with plumieride (25 mg/kg) [25].

#### Group 5:

CA-infected group treated with plumieride (50 mg/kg) [25].

### Sampling

At day one post-infection, skin biopsy was collected from mice to measure the fungal count prior to treatments. Afterwards, all mice were euthanized *via* cervical dislocation on the 4th day to collect skin samples from the affected area. Some specimens were preserved in neutral buffered formalin for 48–72 h to be examined pathologically, while others were maintained at -80 °C for molecular analysis.

### Fungal re-isolation and quantification

The collected fresh samples were homogenized in a stomacher bag containing sterile PBS. Then, 10-fold dilutions of homogenate were plated *via* spiral plater (Autoplate; Advanced Instruments, Inc., Norwood, MA) on nutrient agar at 37 °C for 2 days and sabaroad dextrose agar at 25 °C for 5 days, and then CFU were enumerated.

### Histopathological study

Fixed skin samples were processed through dehydration in graded alcohols, followed by clearance in xylene, and finally embedded in paraffin blocks. Tissue-cut sections of about five microns in thickness were stained with H&E for pathological examination as well as PAS staining for demonstration of *C. albicans* [26]. All sections were examined by an optical microscope (Olympus BX43, Tokyo, Japan) linked to an Olympus DP27 digital camera. The progression of inflammatory reactions and other CA-related skin lesions, including epidermal hyperplasia and edema, was evaluated as follows: (- = nil, < 10% tissue changes), ( + = mild, 10–25% alterations), ( + = moderate, 25–50% alterations), and (+++= severe, > 0% % alterations) [27].

### Immunohistochemical staining and analysis

According to the manufacturer’s procedure, the deparaffinized tissue sections were antigen-retrieved and then blocked by hydrogen peroxide (3%) for twenty minutes. After that, tissue sections were incubated for an hour with anti-iNOS antibody (MAS-16422, Thermofisher Scientific Co., 1:50); then were incubated for twenty minutes with a secondary antibody (HRP Envision kit, DAKO); and treated for ten minutes with diaminobenzidine (DAB). Finally, tissue sections were counterstained with Mayer’s hematoxylin, then dehydrated, cleared, covered, and examined under an optical microscope. At least six random fields were selected from each specimen and analyzed for mean optical density of iNOS immunohistochemical expression by using Leica Microsystems GmbH, Germany, for image analysis.

### Quantitative RT-PCR for the transcriptase levels of TNF-α, IL-1β and NF-κB genes

The target genes’ relative expression in skin tissue is estimated using the quantitative real-time polymerase chain reaction. First, total RNA was extracted from skin samples using the Qiagen mini-RNeasy extraction kit, and the concentration and purity were determined using nanodrops [28]. Next, a Revert Aid First Strand cDNA Synthesis Kit was used to create complementary DNA (cDNA). Primer sets were designed to detect the mRNA levels of the specific genes using Mus musculus sequences from GenBank and the primer3 tool (Table 2). SYBR Green PCR Master Mix was used with the real-time PCR analysis to evaluate the relative gene expression of the target genes, and the ABI Prism Step One Plus Real-Time PCR System was used to run the PCR reactions twice on each sample [29]. An internal control, beta-actin, was employed to normalize the gene expression data. The fold change was calculated using 2-˄˄CT [30].

**Table 2:** The sequence of the primer sets of the target genes

Gene symbol	Gene description	Accession number	Primer Sequence
*TNF-α*	Tumor necrosis factor-α	NM_013693.3	F: 5′- TGTAGCCCACGTCGTAGCAA ‐3′ R: 5′- ATAGCAAATCGGCTGACGGT ‐3′
*IL-1β*	Interleukin-1 β	NM_008361.4	F: 5′- TGCCACCTTTTGACAGTGATG − 3′ R: 5′- AAGGTCCACGGGAAAGACAC − 3′
*NFkb1*	Nuclear factor-κB	AY521463.1	F: 5′- CCCTACGGAACTGGGCAAAT − 3′ R: 5′- TGCAAATTTTGACCTGTGGGT − 3′
*ACTB*	β-actin	NM_007393.5	F: − 5′- CCACCATGTACCCAGGCATT − 3′ R: − 5′- AGGGTGTAAAACGCAGCTCA − 3′

### Statistical analysis of data

An analysis of variance (ANOVA) and Tukey *post-hoc* tests were conducted using the SPSS 25.0 software to determine whether there was a significant difference between the outcomes of the various groups. A statistically significant result was defined as *p* ≤ 0.05. Nonparametric values were analyzed using the Mann-Whitney U test.

## Results

### Antifungal susceptibility testing

The plumieride disk displayed a wider zone of inhibition and lower MIC than FZ disc as shown in Table (3).

**Table 3:** In vitro antifungal activity of fluconazole (FZ) and plumieride on *C. albicans*

Strains		DMSO	FZ	Plumieride
**Clinical isolate**	IZ (mm)	0	12.66 ± 0.57	17.33 ± 2.5
	MIC (µg/mL)	-	128	32
**ATCC 10,231**	IZ (mm)	0	16.33 ± 0.5	21.66 ± 1
	MIC (µg/mL)	-	16	8

Data for inhibitory zone (IZ) are expressed as Mean ± SEM (*n* = 3)

### Multiplex PCR and gene expression

**Fig. 2:**
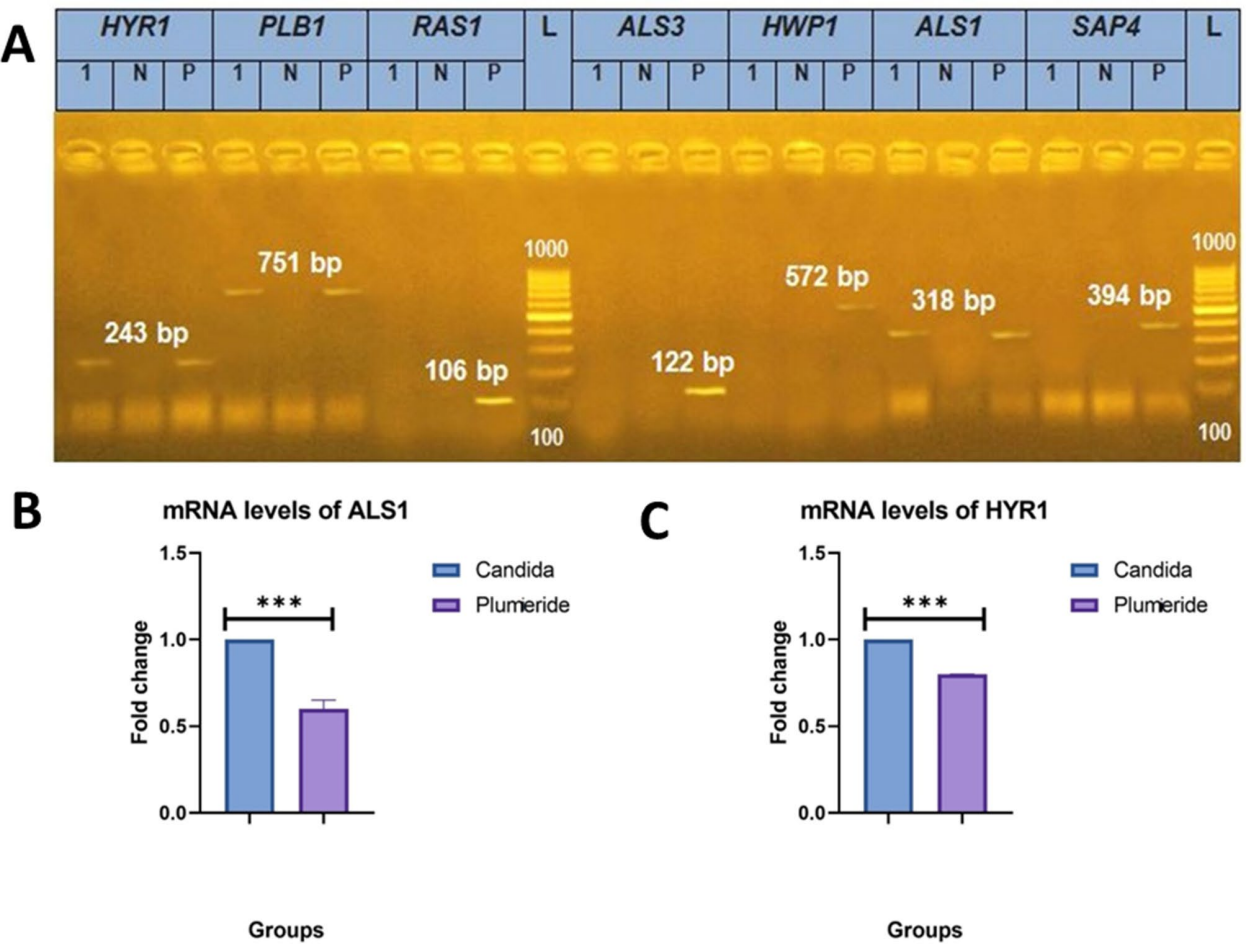
(**A**) Agarose gel photo electrophoresis of conventional PCR on genetic material extracted from *C. albicans* as a molecular typing for detection of SAP4, ALS1, HWP1, ALS3, RAS1, Plb1, Plb1 and Hyr1 genes. Lane L: molecular weight marker (100–1000 BP). Lane Neg: negative control. Lane Pos.: positive control. (**B**) Bar chart showing the fold change of *ALS1* expression upon plumieride treatment. (**C**) Bar chart showing the fold change of *HYR1* expression upon plumieride treatment. Data was expressed as median (*n* = 7) and analyzed using the Mann-Whitney U test. ⁎⁎⁎ means significant difference at 0.001

CA isolate was positive for *PLB1, HYR1, and ALS1* genes, showing bands at 751 bp, 243 bp, and 318 bp, respectively (Fig. 2A). Additionally, *ALS1 and HYR*1 virulence genes were downregulated to 0.64 and 0.8, respectively, upon treatment with plumieride, while the untreated group fold change was 1.0 (Fig. 2B, C).

### Fungal count

At 1-day post-inoculation, the fungal count was the same in all groups. However, in the following days, plumieride reduced the fungal count in a dose dependent manner on the 4th day, in contrast to the CA-infected group. However, the CA-infected mice treated with a high dose of plumieride (50 mg/kg) demonstrated the lowest fungal count (Table 4).

**Table 4:** Fungal count in *C. albicans*-infected mice treated with fluconazole (FZ) and plumieride

	at 1-hr post-inoculation	at 4th day post-inoculation
Non infected	< 100	< 100
CA-infected	6.6 × 10^7^ ± 7.0 × 10^6 a^	4.8 × 10^9^ ± 1.6 × 10^7 a^
CA-infected + FZ	5.8 × 10^7^ ± 1.6 × 10^6 a^	2.5 × 10^2^ ± 28.8^b^
CA-infected + Plumieride (25 mg/kg. bwt.)	4.8 × 10^7^ ± 4.4 × 10^6 a^	2.3 × 10^2^ ± 14.5^b^
CA-infected + Plumieride (50 mg/kg. bwt.)	3.1 × 10^7^ ± 1.6 × 10^6 a^	< 100

Data expressed as Mean ± SEM (*n* = 7), different superscript letters (a, b, c) indicate significance between all *C. albicans* groups at *P* ≤ 0.05

### Histopathological results

**Fig. 3:**
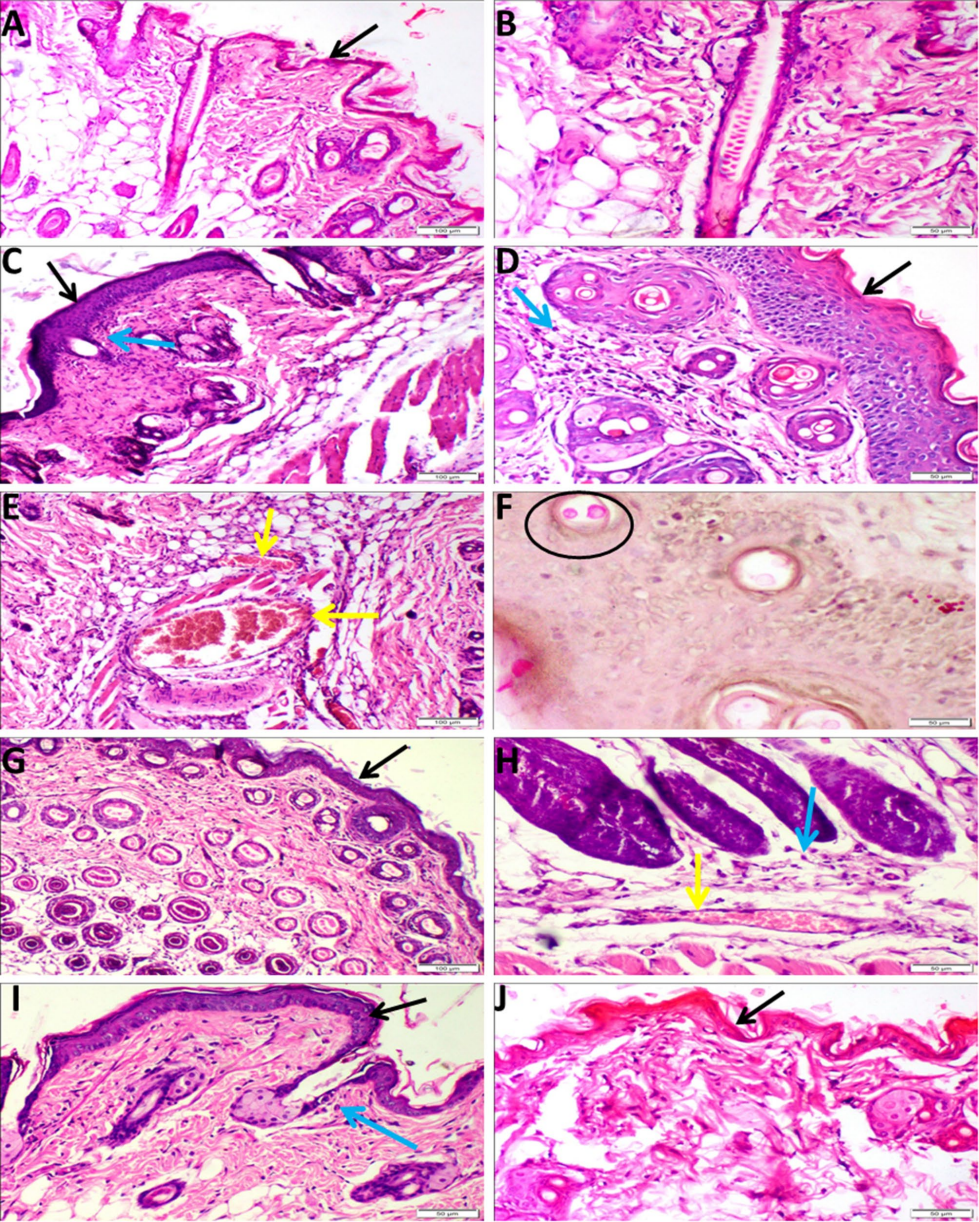
Photomicrograph of skin tissue sections in various groups. (**A**, **B**) Normal control group, (**C** - **F**) *C. albicans*-infected group, (**G**, **H**) FZ-treated group, (**I**) plumieride (25 mg/kg bwt.)-treated group, and (**J**) plumieride (50 mg/kg bwt.)-treated group. Note: Epidermis (black arrows), inflammatory cells (blue arrows), Congested BVs (yellow arrows). All figures are H&E stained except Fig. F is PAS stained for demonstration of *C. albicans* (circle)

Skin samples of the uninfected mice in the control group exhibited normal epidermal, dermal, and hypodermal layers without any histopathological alterations (Fig. 3A and B). On the contrary, CA-infected mice revealed deleterious histopathological changes, including all skin layers. CA caused disorganized thickening of the epidermis, represented by increasing the layers of the stratum spinosum (acanthosis), associated with vacuolar degeneration of epidermal prickle cells (Fig. 3C, D). Multifocal areas of erosion and ulcers were observed in some sections. It also induced an inflammatory process in the dermis characterized by extensive vascular congestion, marked edema and inflammatory cell infiltration (Fig. 3E), especially in the deep dermis and subcutaneous tissue, in which neutrophils are a predominant cell mixed with other lymphoplasmacytic infiltration in addition to the presence of necrotic areas. A decrease in the density of hair follicles and folliculitis were also noticed in CA-infected mice. *C. albicans* (yeast form) was also demonstrated by PAS stain in some examined mice (Fig. 3F). The FZ-treated mice presented an increase in the density of hair follicles in addition to a decrease in the epidermal thickness (Fig. 3G), moderate inflammatory cell infiltration in the dermal layer, and persistence of vascular congestion (Fig. 3H). Skin samples from mice treated with plumieride (25 mg/kg) showed a moderate reduction of epidermal thickness with little inflammatory cell infiltration in the dermal layer and no evidence of vascular congestion (Fig. 3I). However, mice treated with plumieride (50 mg/kg) restored skin nearly to normal (Fig. 3J). The highest score of inflammation, epidermal hyperplasia, and transudation was recorded in the CA-infected group, whereas an observable reduction in the score of all parameters was noticed in both plumieride-treated groups compared to both the CA-infected group and the FZ-treated group (Table 5).

**Table 5:** Semiquantitative microscopic lesion scoring of the skin sections in various groups

	Epidermal hyperplasia	Epidermal vacuolation	Dermal inflammation	Dermal exudation
Non infected	-	-	-	-
CA-infected	+++	+++	+++	+++
CA-infected + FZ	++	++	++	++
CA-infected + 25 mg/kg. bwt. plumieride	+	-	+	-
CA-infected + 50 mg/kg. bwt. plumieride	-	-	-	-

*Notes* (-) nil; (+) mild; (++) moderate; and (+++) severe histopathological changes

### Immunohistochemical analysis

**Fig. 4:**
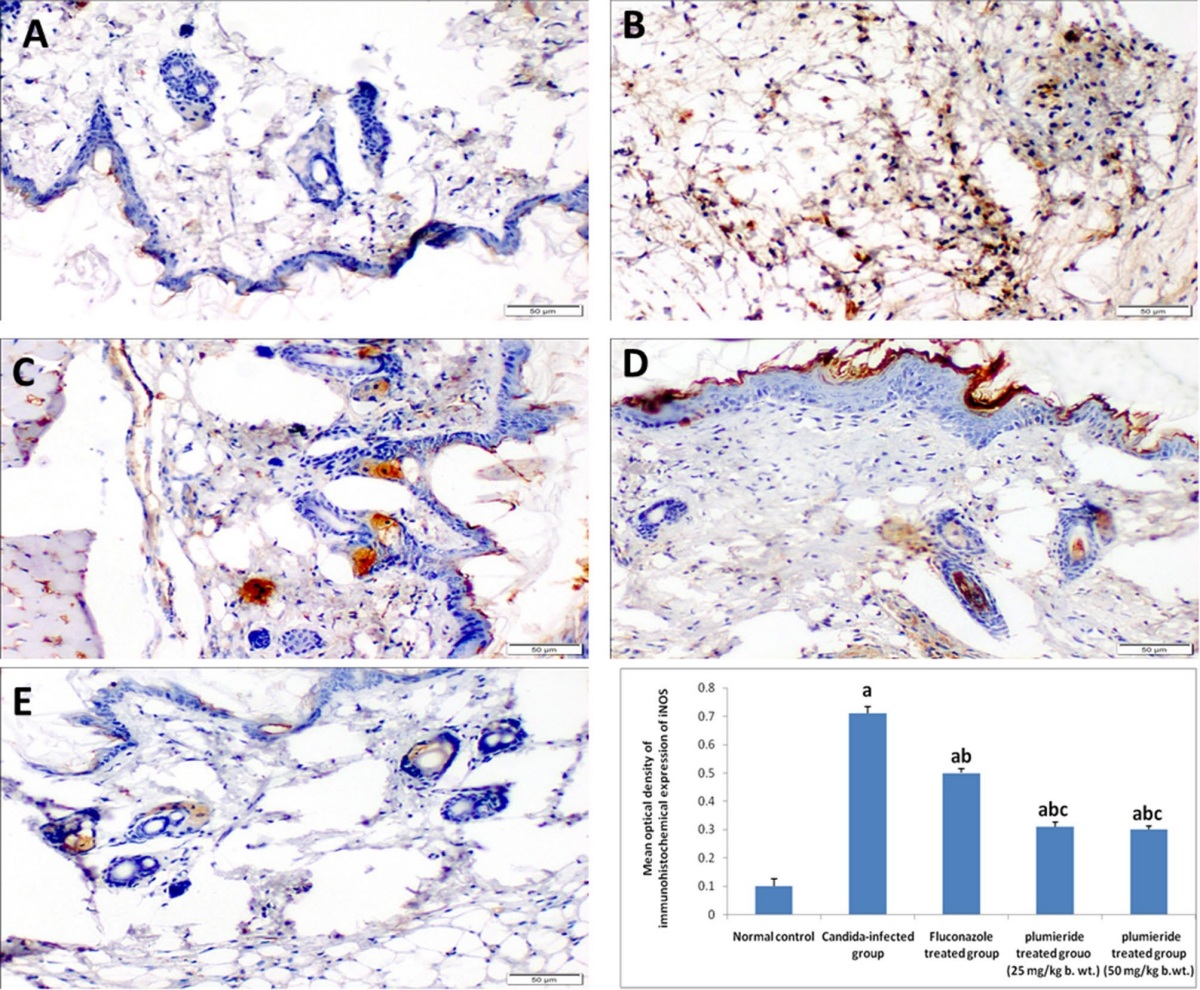
Photomicrograph of skin showing iNOS Immunostaining in diverse groups. (**A**) Normal control group, (**B**) *C. albicans*-infected group (**C**) FZ-treated group, (**D**) plumieride (25 mg/kg bwt.)-treated group, (**E**) plumieride (50 mg/kg bwt.)-treated group. Data represented as mean ± SEM (*n* = 7). Different superscript letters indicate significant difference at *P* ≤ 0.05

As shown in Fig. 4, the CA-infected group revealed strong positive immunohistochemical expression of iNOS (mean optical density of 0.71 ± 0.02) compared to that of the control non-infected group (0.1 ± 0.02). However, the FZ-treated group demonstrated a moderate expression of iNOS (the optical density of 0.50.01). In contrast, a significant decrease in the expression of iNOS was observed in the skin tissue samples of both plumieride-treated groups (0.3 ± 0.01) compared to that of the CA-infected group.

### Quantitative RT-PCR for the transcriptase levels of *TNF-α*, *IL-1β* and *NF-κB* genes

**Fig. 5:**
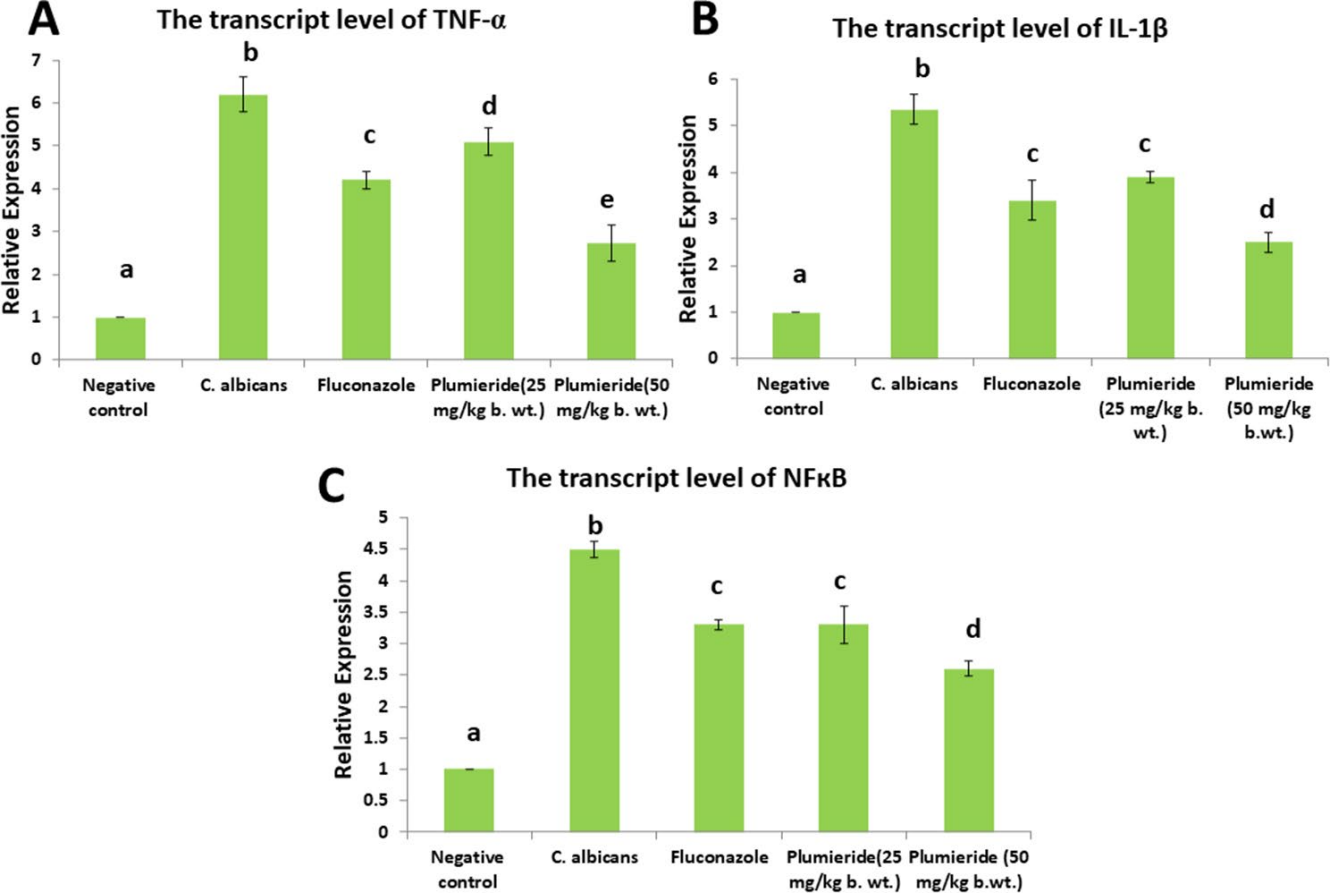
Bar chart representing the transcript levels of (**A**) TNF-α, (**B**) IL-1β (**C**) NF-κB. Values are presented as mean ± SEM (*n* = 7). Different superscript letters indicate a significant difference at *P* ≤ 0.05

The transcript levels of *TNF-α*, *IL-1β*, and *NF-κB* recorded a significant upregulation in the CA-infected group. The pro-inflammatory genes were significantly downregulated in all treated groups, mainly in the plumieride-treated group (50 mg/kg) (Fig. 5).

## Discussion

Superficial *Candidi*asis is a common nosocomial infection that spreads quickly among hospitalized people around the world [31]. Although fluconazole is the antifungal of choice in many cases, its administration is often linked to the risk of kidney damage at first, and the prolonged use of FZ is accompanied mostly by the emergence of FZ-resistant strains [32]. These factors can result in treatment failure and potentially serious complications. Accordingly, there is a need for ongoing research and innovation in antifungal therapy. Several natural products from the Middle East were reported to have anti-*Candida* activities, but none of them have ever been clinically used to treat fungal skin infections [33]. *Plumeria obtusa* L. is commonly cultivated in this region for ornamental purposes, and infusions of the leaves are traditionally used to treat wounds and skin conditions [34]. Also, the plant extracts demonstrate a broad spectrum of biological activities, such as anti-inflammatory, antioxidant, and antibacterial activities [35]. To the best of our knowledge, this study is the first to demonstrate the effectiveness of one of the major iridoids, plumieride, against cutaneous candidiasis compared to the standard fluconazole.

*C. albicans* has multiple strong cell wall proteins called glycosyl phosphatidyl inositol (GPI)-anchored proteins, which are major contributors to fungal virulence. The adhesion/invasion of host tissues, as well as the ability to evade the host immune defense, are major virulence factors expressed by proteins from the agglutinin-like sequence family and hyphal-regulated-like genes, respectively. The N-terminal of these proteins is mostly conserved and forms a β-helical structure, like bacterial adhesin factors. The screening of virulence genes showed that the CA isolate tested positive for *ALS*, *PLB1*, and *HYR1* genes. Accordingly, the isolate is regarded as highly virulent and resistant to multiple drugs [36, 37]. Based on qRT-PCR analysis, it was observed that plumieride treatment resulted in a significant decrease in the expression levels of hypha-specific genes (such as *HYR1*) and biofilm genes (*ALS1*) in CA-infected mice. Specifically, the expression of *HYR1* was downregulated by nearly 20%, while the expression of *ALS1* was downregulated by approximately 35% in the presence of plumieride. These findings suggest that plumieride has the ability to inhibit CA morphogenesis by suppressing the expression of genes associated with hyphal growth and biofilm formation. Therefore, plumieride is thought to have a potent anti-virulent effect against CA.

In the current study, the antifungal activity of plumieride vs. FZ was investigated both in vitro and in vivo. The in vitro susceptibility studies revealed that the isolate was sensitive to plumieride disc, as demonstrated by a wider inhibition zone and lower MIC compared to FZ disk. Likewise, the in vivo study proved that plumieride showed superior antifungal activity compared to fluconazole against cutaneous candidiasis in a murine model. Plant iridoids were reported as potential antimicrobial (antibacterial, antifungal, and antiviral) agents [38]. Genus *Plumeria* is known as a source of iridoids. Furthermore, four iridoids (plumieride A, B, and C and epiplumeridoid C) previously isolated from the stem bark of *P. rubra* were reported to exhibit promising antifungal and antibacterial activities [39]. Plumieride, previously isolated from the aqueous extract of *Allamanda polyantha* seeds, demonstrated antifungal activity against *Cryptococcus* spp. and caused morphological alterations in the C. *neoformans* H99 strain [40]. Also, it demonstrated antifungal activity against dermatophytes and phytopathogens, and immunostimulatory activities [41–44]. Furthermore, plumieride inhibited the radial mycelial growth of *Phomopsis vexans, Phytophthora capsici, Fusarium oxysporum, Rhizoctonia solani*, and *Sclerotium rolfsii in vitro* [43]. It has been suggested that the antimicrobial properties of *Plumeria* flowers can be attributed to the presence of various phytochemical compounds with anti-inflammatory, anti-quorum sensing, and antioxidant properties [45].

In the current in vivo study, the histopathological examination of the CA-infected mice revealed dermatitis and hyperplastic epidermis. The progressive infiltration of the dermis by mononuclear inflammatory cells could be attributed to the ability of *C. albicans* to stimulate the immune response in the tissue by promoting the expression of proinflammatory cytokines, as previously reported  [46]. Also, the observed epidermal hyperplasia plays a vital role in protecting the body from external infections and maintaining the integrity of the skin [47]. The observed inflammatory reactions were confirmed by immunohistochemical staining and RT-PCR analysis of some inflammatory genes. The CA-infected mice demonstrated a strong positive expression of iNOS. Previous research has reported that iNOS expression is stimulated by IL-1β [48]. Additionally, cytokines such as IL-1β and TNF-α have been found to upregulate iNOS in mice [49], which suggests that iNOS higher expression in candidiasis is mediated by IL-1β and TNF-α. These findings are in line with our results about the upregulation of TNF-α and IL-1β and NF-κB genes in the *Candida*-infected group confirming its induction of an inflammatory process. Similarly, Schaller et al. found that the epithelial cells produced a significant amount of TNF-α and IL-1β during the vaginal *C. albicans* infection. The IL-1β family is known for its powerful inflammatory modulating properties [46]. IL-1β, through its membrane receptor, initiates a signal transduction cascade that activates NF-κB [50]. NF-κB activation can then regulate and initiate the expression of a number of inflammatory cytokines that play a role in the inflammatory process [51].

On the other hand, plumieride treatment was found to reverse the histopathological changes in the skin tissue caused by CA. It also prevents the increase in iNOS expression and reduces the levels of proinflammatory genes like TNF-α, IL-1β, and NF-κB. These findings suggest that plumieride may have potential therapeutic effects in treating candidiasis *via* its anti-inflammatory activity. Iridoids and secoiridoids in *Plumeria* plants were reported to have anti-inflammatory, antioxidant, antibacterial, and antifungal activities [52]. Lotankar et al. (2016) showed that the ethanolic extract of *P. obtusa* markedly reduced the inflammation in carrageenan-induced toxicity in rats [53]. Another study reported that aerial parts of *Plumeria obtusa* have anti-inflammatory activity against lipopolysaccharide-induced pneumonia in mice through their ability to decrease the release of inflammatory cytokines [12]. Boeing et al., proved that plumieride could attenuate ulcerative colitis through its strongest antioxidant and anti-inflammatory effects [54].

## Conclusions

Plumieride has potential antifungal activity against *C. albicans* growth and colonization. Its comparable mechanism of action to fluconazole and its ability to reduce candidiasis-induced skin damage in the infected mice are promising findings and suggest that plumieride is a potential treatment option for superficial candidiasis. Additionally, the development of an anti-fungal cream containing plumieride could be explored to enhance its effectiveness in treating this condition.

### Electronic supplementary material

Below is the link to the electronic supplementary material.

Supplementary Material 1

## Data Availability

The authors declare that the data supporting the findings of this study are available within the paper. Should any raw data files be needed in another format they are available from the corresponding author upon reasonable request. Source data are provided with this paper.

## Abbreviations

MIC: Minimum inhibitory concentration
qRT-PCR: Quantitative real-time -polymerase chain reaction
TNF-α: Tumor necrosis factor-alpha
IL-1β: Interleukin-1 beta
NF-κB: Nuclear factor kappa B
iNOS: Inducible nitric oxide synthase
*ALS*1: Agglutinin-like sequence gene
*Plb*1: Phospholipase B
*Hyr*1: Hypha-specific gene
