# Supplementary material for: Plumieride as a novel anti-fungal and anti-inflammatory iridoid against superficial candidiasis in mice
Source: BMC Complement Med Ther. 2024 Jun 10;24:224. doi: 10.1186/s12906-024-04508-z (PMC11163697; doi:10.1186/s12906-024-04508-z)
Supplement: Supplementary file 1 — Supplementary Material 1 [file 12906_2024_4508_MOESM1_ESM.pdf]

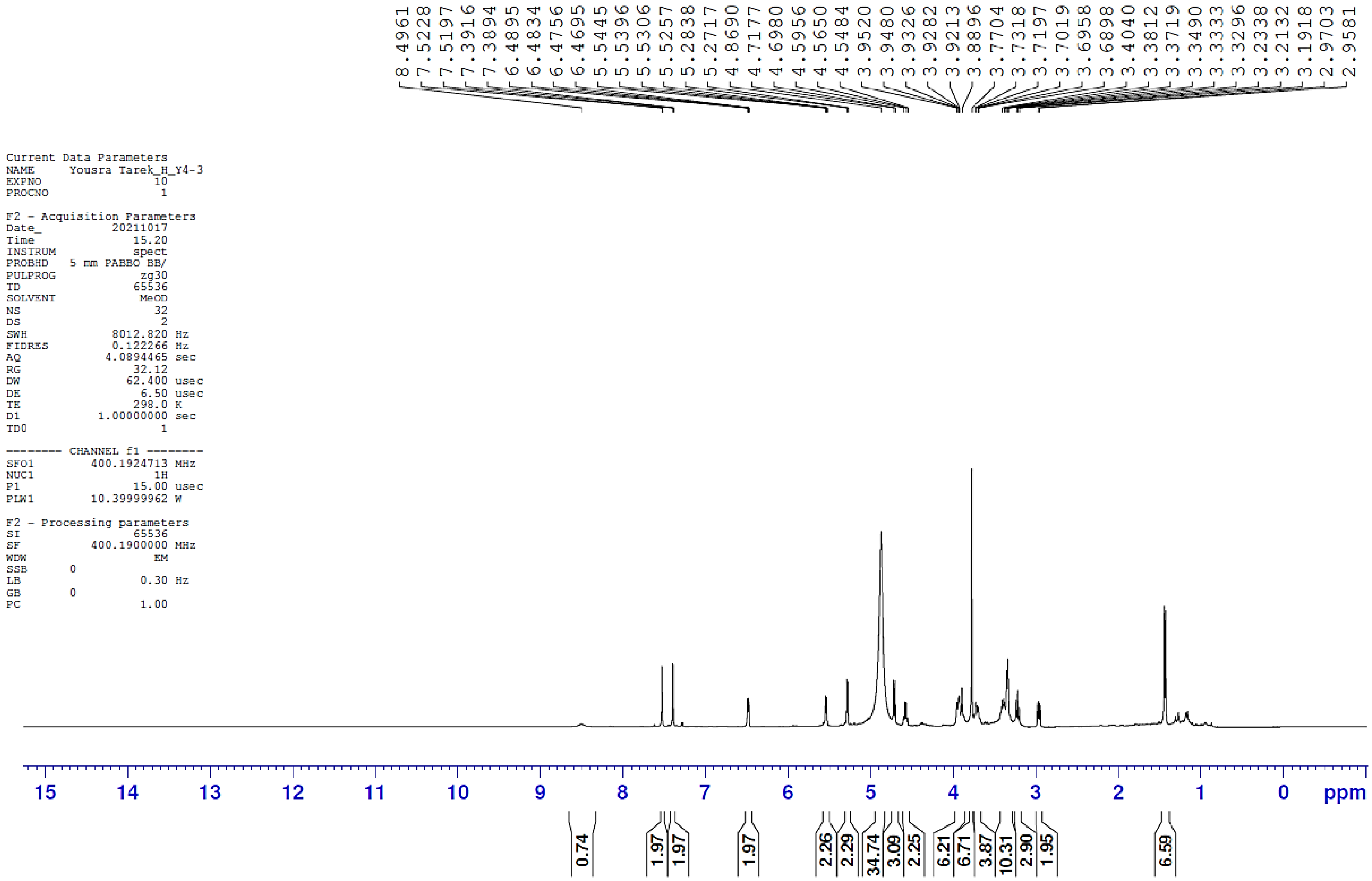

Fig. S1. ^{1}H-NMR of plumieride.

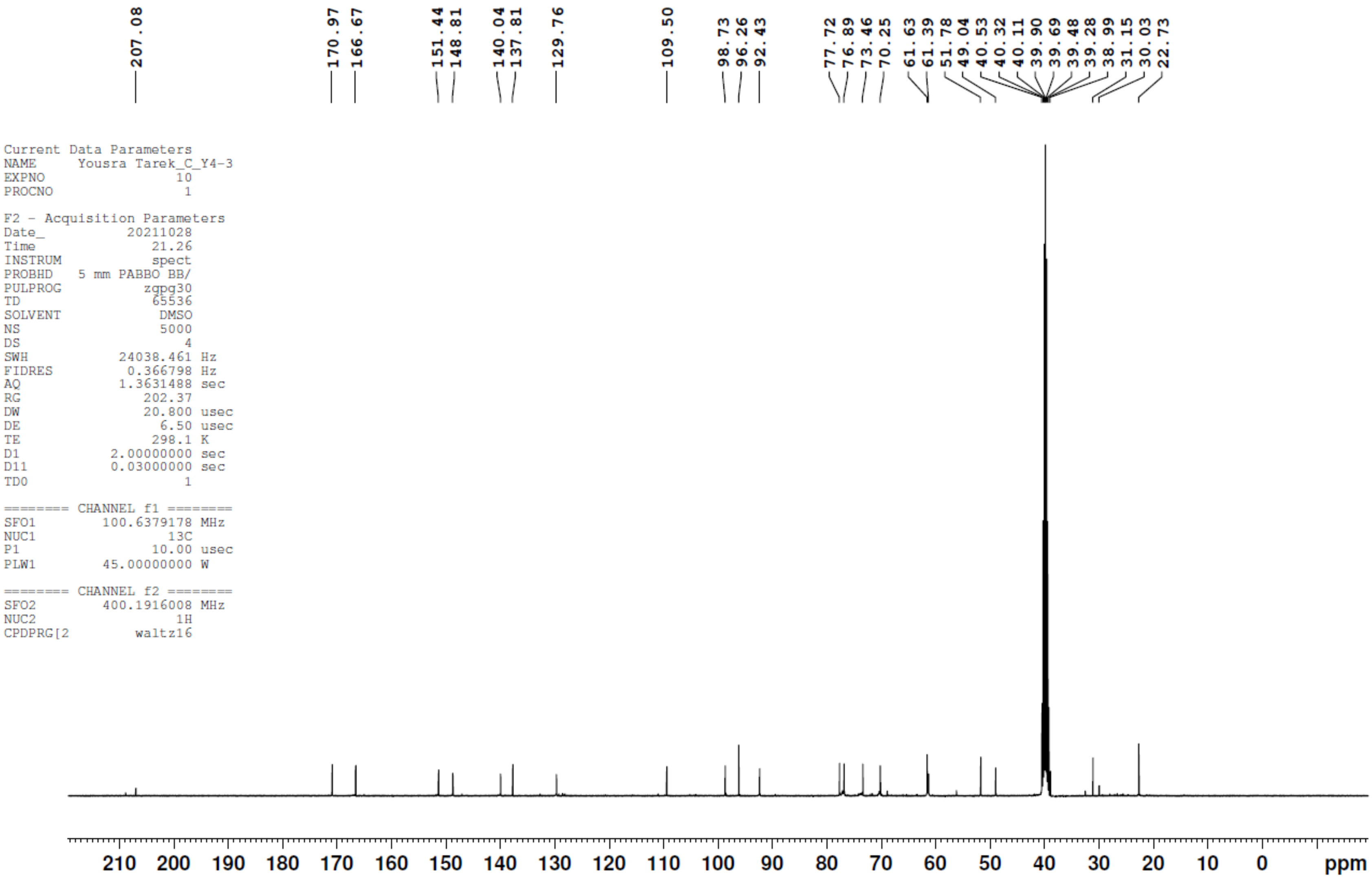

Fig. S2. ^{13}C-NMR of plumieride.

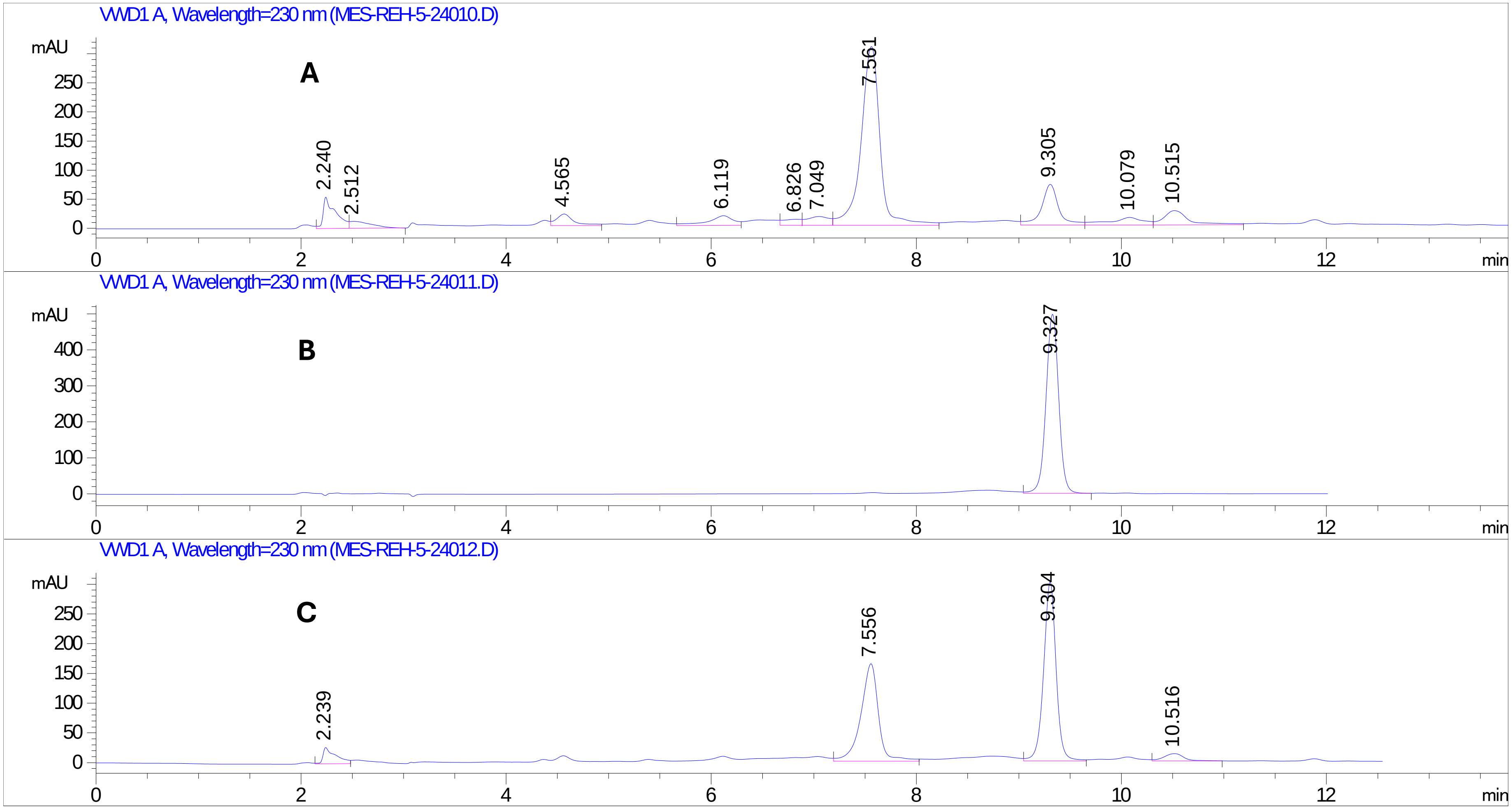

Fig. S3. (**A**) HPLC chromatogram of the methanolic fraction of *P. obtusa* (2 mg/ mL), (**B**) HPLC chromatogram of the isolated compound (plumeiride, 1mg/ mL), and (**C**) HPLC chromatogram of the methanolic fraction (1 mg/ mL) + plumeiride (0.5 mg/ mL) applying spiking technique.
